# Increased Vδ1γδT cells predominantly contributed to IL-17 production in the development of adult human post-infectious irritable bowel syndrome

**DOI:** 10.1186/s12876-021-01722-8

**Published:** 2021-06-30

**Authors:** L. W. Dong, X. N. Sun, Z. C. Ma, J. Fu, F. J. Liu, B. L. Huang, D. C. Liang, D. M. Sun, Cheng Lan

**Affiliations:** 1 Department of Gastroenterology, Hainan General Hospital, Affiliated Hainan Hospital of Hainan Medical University, Haikou, 570311 Hainan Province China; 2 Doheny Eye Institute, Department of Ophthalmology, David Geffen School of Medicine, University of California, Los Angeles,, Los Angeles, CA 90033 USA

**Keywords:** Post-infectious irritable bowel syndrome, Γδ T cells, Subset, Function, Pathogenesis

## Abstract

**Background:**

γδT cells play an important role in the mucosa inflammation and immunity-associated disorders. Our previous study reported that γδ T cells producing IL-17 were involved in the pathogenesis of post-infectious irritable bowel syndrome (PI-IBS). However, their subset characteristic profile in this kind of disease remains unclear. Thus the current study’s aim is to investigate the functionally predominant subset and its role in PI-IBS.

**Methods:**

The total T cells were collected from the peripheral blood of patients with PI-IBS. The peripheral proportion of Vδ1 and Vδ2 subset was detected by FACS after stained with anti δ1-PE and anti δ2-APC. The local colonic proportion of this two subsets were measured under laser confocal fluorescence microscope. Vδ1 γδ T cells were enriched from the total peripheral T cells by minoantibody-immuno-microbeads (MACS) method and cultured, functionally evaluated by CCK-8 assay (proliferation), CD69/CD62L molecules expression assay (activation) and ELISA (IL-17 production) respectively.

**Results:**

1. Vδ1 γδ T cells significantly increased while Vδ2 γδ T cells remained unchanged in both the peripheral blood and local colonic tissue from PI-IBS patients (*p* < 0.05). 2. When cultured in vitro, the Vδ1 γδ T cells remarkably proliferated, activated and produced IL-17 (*p* < 0.05).

**Conclusions:**

Our results suggest that Vδ1 γδ T cells was the predominant γδ T cells subset in both peripheral and intestinal tissue, and was the major IL-17 producing γδ T cells in PI-IBS.

## Background

Post-infectious irritable bowel syndrome (PI-IBS) is a kind of functional gastrointestinal disorders, with clinical feature of abdominal pain or discomfort accompanied with abnormal defecate habit and /or stool character. The incidence of this disease ranges from 10 to 60%, predominantly in the developed western industrialized countries. During the last three decades, it is reported to occur in the developing countries [1]. Irritable bowel syndrome (IBS) which occured after an initial episode of acute gastrointestinal infection was defined as postinfectious irritable bowel syndrome (PI-IBS) [2]. Suffering from the refractory symptoms, the patients are living with low life quality and have to be charged with expensive medical bills. Without obvious morphological changes and biochemistry abnormity, this kind of disease is short of specific and effective therapy [3]. The pathophysiological mechanisms of PI-IBS lie in its persistent low-grade inflammation in the intestines. Evidences suggest that there is low-grade inflammation in the colonic mucosa and/or a state of immune activation in patients with irritable bowel syndrome (IBS) [4]. Basically, it is regarded as a post-inflammatory immune disturbance [5].

Regulation of inflammatory response might contribute to alleviate symptom of PI-IBS. Research reported that EphA2, a member of Eph receptor family, could regulate inflammation and oxidative stress via Nrf2 and NF-Κb signaling pathway. Upregulating of EphA2 and activation of NF-Κb signaling pathway exerts beneficial effect in PI-IBS [6]. Moreover, non-coding RNAs also showed the pivotal role in the regulation of inflammatory response in PI-IBS. Zhang et al. found that miRNA-510, a widely investigated miRNA in cancer progression, downregulated in intestinal tissue might contribute to inflammatory injury and PI-IBS via targeting PRDX1 [7]. Accounting for only 0.5–5%of the whole T cells, γδT cells are located in peripheral and local mucosa-associated lymphoid tissue (MALT), such as derma, respiratory tract, reproductive tract and digestive tract, recognizing the pathogens directly or presenting antigen to effect cells like B cells, thus triggering the specific immune response [8–11]. In some disorders, γδT cells are proved to be the major resource of IL-17 [12–15]. Additionally, IL-17 is also involved in the development of PI-IBS. For instance, Rifaximin could alleviated visceral hypersensitivity, recovered intestinal barrier function, and inhibited low-grade inflammation in colon and ileum of PI-IBS mouse model via suppressing the expression of IL-17 and promoting the expression of the major tight junction protein occluding [16].

It has been proved that γδT cells are involved in the pathogenesis of experimental autoimmune encephalomyelitis, rheumatoid arthritis, Non-obese diabetes [17–19]. On the other hand, their protective role was observed in rats with colitis and sepsis, and mouse with experimental autoimmune uveitis [20, 21].

Recently, the role of the γδT cells’ subset in the diseases is becoming a research hotspot. The γδTCR is constituted by γ and δ variable regions. The Vγ and Vδ genetic locus possess recombinant multiformity, which include γ1–γ9 and δ1–δ3 variable regions. According to the various expression of V gene segments, γδ T cells can be divided into various subsets, the heterogeneity of which determinates their functional diversity. Vδ1 γδ T cells is mostly located in the thymus and mucosa epithelium, while a small amount exist in the peripheral blood. Vδ1 γδ T cells recognize the stress molecule by its TCR and activate as a producer of some pro-inflammatory cytokines to triger the fast immune response. For example, Vδ1 γδ T cells produce IL-17 in pneumococcus infection and protect mice from Listeria infection [22, 23]. Vδ2 γδ T cells are the major γδ T cells in the peripheral circulation of healthy human, accounting for 50%-90% of the total γδT cells, presenting the antigen to the B and NK lymphocytes and producing IFN-γ [24, 25]. Vδ3 γδ T cells are mainly enriched in the liver, accounting for the minimum of the total γδ T cells (0.2%) [26]. We previously reported that the intestinal γδ T cells could exert an important role in a PI-IBS mouse model [27]. However, the precise mechanism of γδT cells subset in this kind of disease remain unclear, thus the current study aims to investigate the changes of the γδT cells subsets and its functional meaning in PI-IBS.

## Methods

### PI-IBS patients

From January 2018 to December 2019, thirty-five IBS patients including inpatients and outpatients were recruited for the study at Hainan General Hospital. The inpatients met the criteria that having abdominal pain, diarrhea, or abdominal pain accompanied by changes in stool characteristics. No obvious biochemical or pathological abnormalities were observed after multiple examinations, including colonoscopy. All hospitalized patients excluded other organic diseases and were clearly diagnosed with irritable bowel syndrome. All 35 patients met the Rome III dignosis criteria for IBS and were positively confirmed onset after an episode of acute gastroenteritis with diarrhea and/or vomiting in addition to a series of examinations such as microbiology test, hence, meeting the definition of PI-IBS [28]. In PI-IBS group, we performed two consecutive stool cultures and fungal examinations on all patients, and only those with negative results can be included in the group. All patients underwent colonoscopy. There was no obvious inflammation under the microscope, and there were a few chronic inflammatory cells in histological examination. Moreover, microscopic colitis and other inflammation GI diseases were excluded by histological screening of mucosal biopsies throughout the entire colon, obtained by colonoscopy prior to inclusion according to established diagnostic criteria. The exclusive criteria for patients were as follows: (1) experienced major abdominal surgery. (2) Evidence of metabolic, gastrointestinal, cardiovascular, psychological or malignant disease. (3) Gastrointestinal organic disease including peptic ulcer (all patients tested by upper GI endoscopy), Crohn’s disease, ulcerative colitis and pancreatitis (all patients tested by blood amylase examination and abdominal ultrasound examination). (4) Pregnancy or lactating. (5) Patients who are taking nonsteroidal anti-inflammatory drugs, steroids, or antibiotics. The patients’ age ranged from 17 to 53 years old, with the mean age of 33.9 years, including 19 female and 16 male. In this study, 33 voluntary healthy controls were recruited by advertisement and had regular examination including colonoscopy to exclude any inflammatory diseases. None of them had GI complaints, chronic pain conditions, infectious or inflammatory disorders such as rheumatoid arthritis, psychiatric illnesses, or were taking pharmaceutical agents. The age of health controls ranged from 19 to 55 years old, with the mean age of 32.9 years, including 15 female and 18 male. The peripheral blood and colonic tissue were collected through colon biopsy and preserved in − 80 °C freezer for further examination. In addition, some tissue was processed into frozen sections. Some tissue was smashed into powder within ultrosonic disintegrator and the supernatant was preserved under − 80 °C freezer for further examination.

### Morphological analysis

The colonic tissue was collected by colonoscopy biopsy and processed into ultra-thin frozen sections by liquid nitrogen quick-frozen slicing. The sections were treated by immunofluorescence histochemical staining. The primary antibody was florescence- conjugated rabbit anti-mouse anti-δ1 TCR or δ2 TCR monoclonal antibodies. The stained tissue sections were scanned under Laser scanning confocal microscope (Olympus FV10i, Olympus, Tokyo, Japan). The intensity of fluorescence was calculated automatically using the image analysis software.

### Enrichment of Vδ1 γδ T cells

The enrichment of T cells were conducted as previously described by Cheng et al. [11]. Briefly, 5 ml peripheral blood of PI-IBS patients was collected and the total T cells were isolated by centrifuging with lymphocyte separation medium Ficoll (Sigma-Aldrich, ST. Louis, MO, USA). The total T cells were stained with anti humanVδ1-PE and anti humanVδ2-APC (BD Biosciences, Franklin Lakes, NJ, USA) and the proportion of Vδ1 and Vδ2 γδ T cells was measured by FACS. In conclusion, the proportion of Vδ1 T cells were all increased in 35 PI-IBS patients compared with control group.

The total T cells were stained with FITC conjugated anti-δ1 TCR mAb (100ul antibodies per 1 × 10^8^ cells, incubated at 4 °C for 30 min), followed by stained with microbeads conjugated anti-FITC mAb (100 ul antibodies per 1 × 10^8^ cells, incubated at 4 °C for 30 min). The Vδ1 γδT cells were positively selected by MACS (Miltenyi Biotec GmbH, Cologne, NRW, Germany). For further purification of Vδ1 γδ T cells, the residual αβ+ T cells were depleted using PE-conjugated anti-αβTCR antibody with anti-PE microbeads.

### Functional evaluation of Vδ1 γδ T cells

The enriched Vδ1 γδ T cells were incubated in the presence of IL-23 (10 ng/ml) under 37 °C, 5%CO_2_ for 48 h, followed by functional evaluation including proliferation, activation and cytokine producing capability.

#### Proliferation

Cell Counting Kit-8 (CCK-8) Assay was used to evaluate the enriched Vδ1 γδ T cells’ proliferation. Briefly, the cells were seeded at 4 × 10^3^ cells/well in 96-well plates, then incubated at 37 °C for 24 h in a total volume of 100 μl medium. The cells were added with 10 μl CCK8/well and cultured at 37 °C, 5% for 8 h. The absorbance value at 450 nm was measured on a Thermomax Microplate Reader (Menlo Park, CA, U.S.A). The proliferation response was expressed as the OD value of mean ± standard deviation (SD) of triplicate determinations.

#### Activation

Vδ1γδT cells were stained with PE conjugated anti-CD69 mAb or anti-CD62L mAb (100ul antibodies per 1 × 10^8^ cells, incubated with IL-23 and TLR4 at 4 °C for 30 min). The expression of CD69 and CD62L on Vδ1γδT cells was determined by FACS as described previously [11, 14].

#### Production of proinflammatory cytokines

The concentration of IFNγ and IL-17 in the supernatants from the cultured Vδ1 γδ T cells and the colonic tissue were measured by ELISA in accordance with the manufacture’s instruction. Briefly, the supernatant from cultured cells and tissues were removed and replaced with fresh media (RPMI-1640) and were then returned to standard culture conditions for 24hours. Subsequently, the IL-17 protein of cell supernatant was analyzed by IL-17 high-sensitivity (0.25–16 pg/ml sensitivity range) ELISA kit (R&D Systems, Minneapolis, Minnesota, USA) according to the manufacturer’s protocol.

### Statistics analysis

Experimental data were analyzed with Kolmogorov–Smirnov test by SPSS software to explore whether data complied with a normal distribution. The result of K-S analysis indicated a normality distribution of all data. The unpaired Student’s t-test was used to compare differences between two groups (SPSS 19.0 software). Data were expressed as the mean ± standard error of the mean. Values in the same row with different superscripts are significant (*p* < 0.05), while values with same superscripts are not significant (*p* > 0.05).

## Results

### Proportion of γδ T cells subset

#### Proportion of γδ T cells subset in peripheral blood from PI-IBS patients

**Fig. 1:**
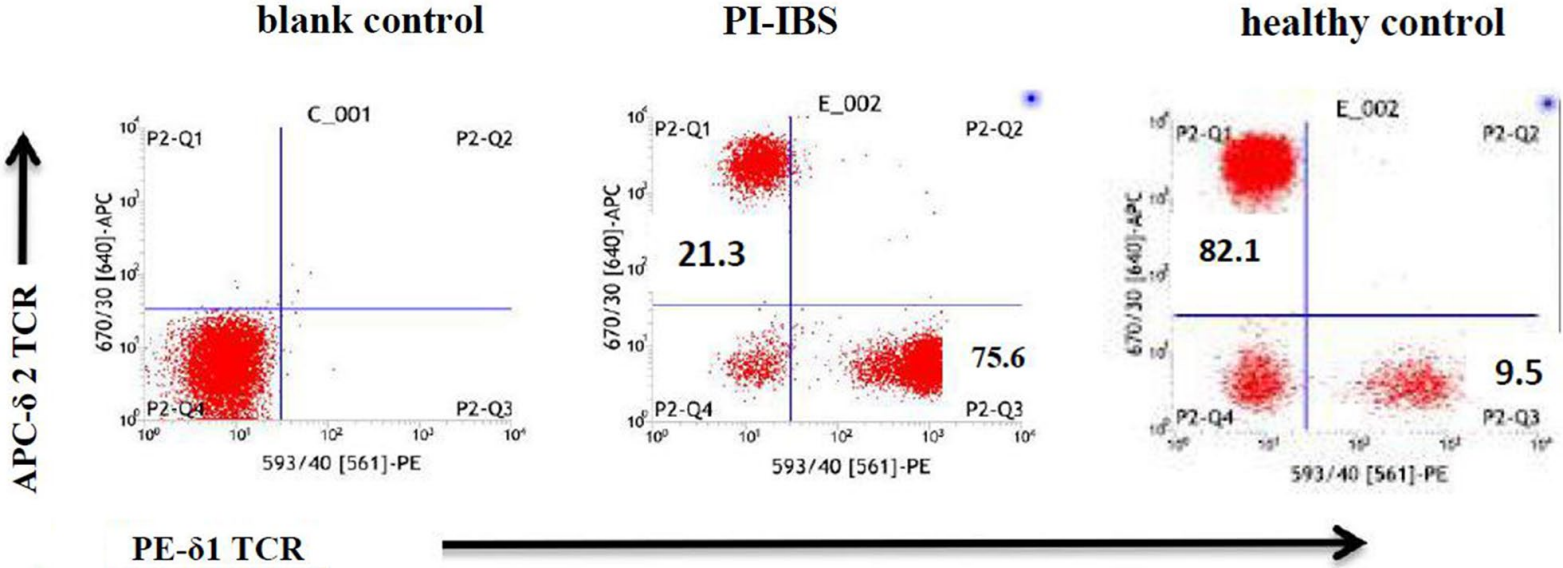
Proportion of γδ T cells subset in peripheral blood from PI-IBS patients was detected by FACS (PE- δ1TCR/APC- δ2TCR). The results indicated that Vδ1 γδT cells significantly increased and became the predominant subset compared with heathy controls, while Vδ1 γδ T cells decreased and became the minor subset in PI-IBS patients, *p* < 0.01. Representative graph were shown in the current figure

**Figure2:**
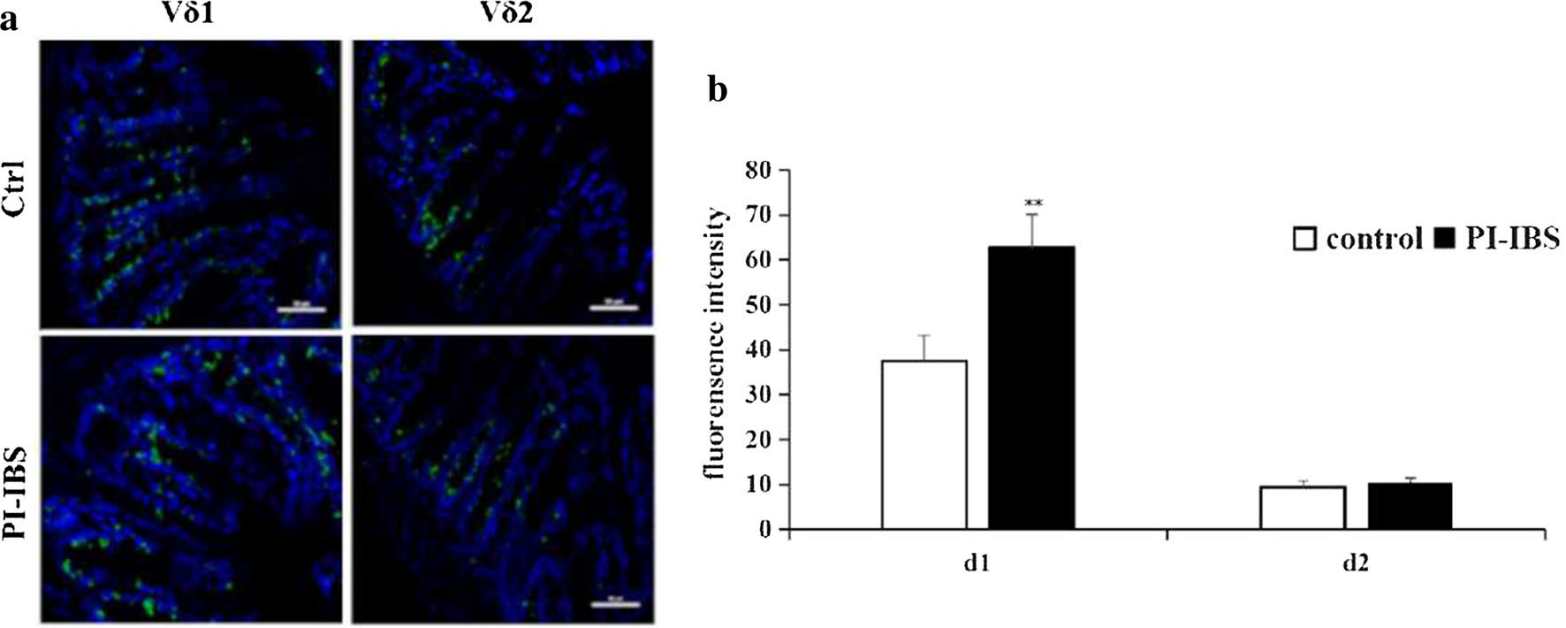
**a** The colonic tissue was collected from PI-IBS patient by colonic endoscopic biopsy and processed into frozen sections, followed by immunofluorescent staining with anti-δ1/δ2 monoclonal antibody. Under laser scanning confocal microscope (200×), the intestinal Vδ1 γδ T cells from PI-IBS patients significantly increased while Vδ2 γδ T cells decreased compared with that from control group, *p* < 0.01. Representative graph were shown in the current figure

Proportion of γδ T cells subset in peripheral blood from PI-IBS patients was detected by FACS (PE-δ1TCR/APC-δ2TCR). Usually, Vδ1 γδ T cells dominate in local mucosa tissue and Vδ2 γδ T cells were the major peripheral γδ T cells. However, in PI-IBS patients’ peripheral blood, Vδ1 γδ T cells significantly increased and became the predominant subset, while Vδ2 γδ T cells decreased and became the minor subset (Fig. 1, Table 1). The intestinal Vδ1 γδ T cells significantly increased while Vδ2 γδ T cells remained 155 unchanged (Fig. 2). The results suggest that the γδ T cells’ characteristic subset profile shifting from Vδ2 to Vδ1 could have potential functional significance.

**Table 1:** Proportion of γδ T cells subset in peripheral blood from PI-IBS patients

	Number	Vδ1 (%)	Vδ2 (%)
Control	n = 33	12.46 ± 2.10	76.87 ± 7.93
PI-IBS	n = 35	80.18 ± 6.24^a^	17.48 ± 3.18^b^

^a^Compared with control group, t = 47.86, *p* = 0.000;

^b^Compared with control group, t = 35.73, *p* = 0.000

#### Enrichment and identifying of Vδ1 γδ T cells

**Fig. 3:**
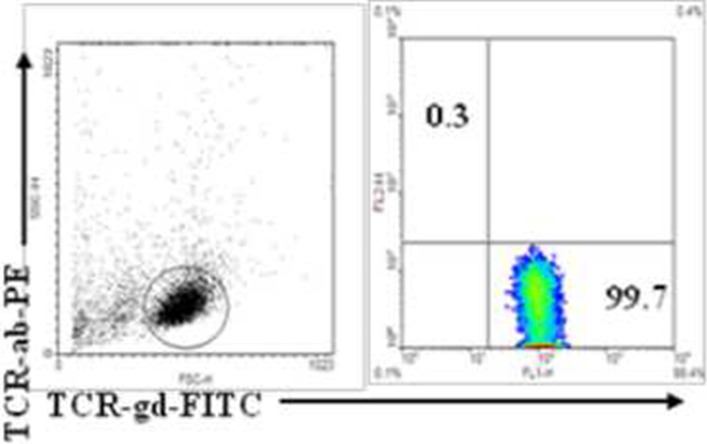
The Vδ1 γδ T cells were isolated by positively selection protocol (MACS) from the total T cells from the peripheral blood of the PI-IBS patients, the purity degree up to 99.7%

The Vδ1 γδ T cells were isolated by positively selection protocol (MACS) from the total T cells from the peripheral blood of the PI-IBS patients. After the contaminant αβ T cells were depleted, the proportion of the purified Vδ1 γδ T cells was up to 99.7% (Fig. 3). And the cell viability remained up to more than 90%. Thus the high degree of purity of the enriched Vδ1 γδ T cells guaranteed their functional analysis in vitro.

### Functional analysis of Vδ1 γδ T cells

**Fig. 4:**
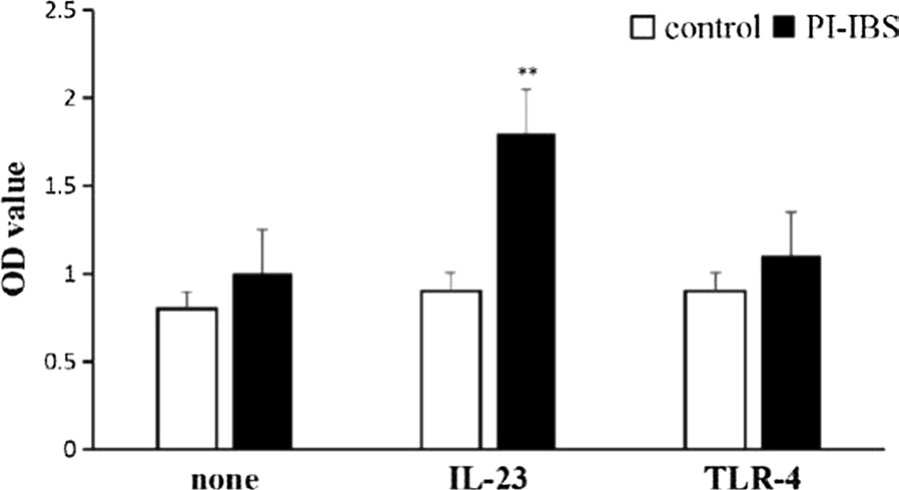
CCK-8 Assay was used to evaluate the enriched Vδ1 γδ T cells’ proliferation stimulated with IL-23 or TLR-4. As shown in Fig. 4, compared with the control group, in the presence of IL-23, the Vδ1 γδ T cells from PI-IBS patients significantly proliferated. All experiments were repeated three times. *p < 0.05, **p < 0.01

**Fig. 5:**
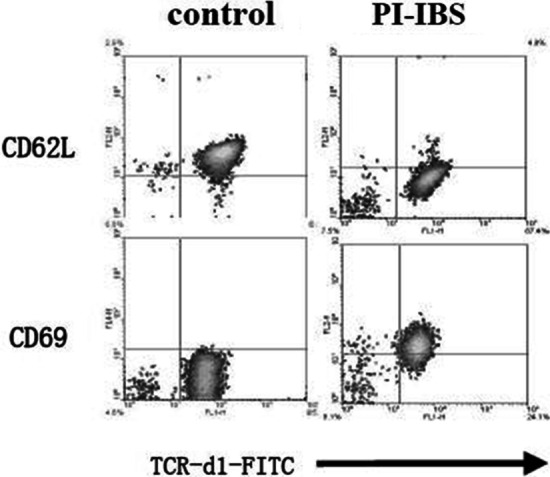
The expression of CD62L and CD69 molecules on the enriched Vδ1 γδ T cells were detected to evaluate the activation status of the subset. Expression level of CD62L remarkably decreased while that of CD69 increased on the surface of Vδ1 γδ T cells from PI-IBS patients compared with heathy controls. All experiments were repeated three times. *p < 0.05, **p < 0.01

**Fig. 6:**
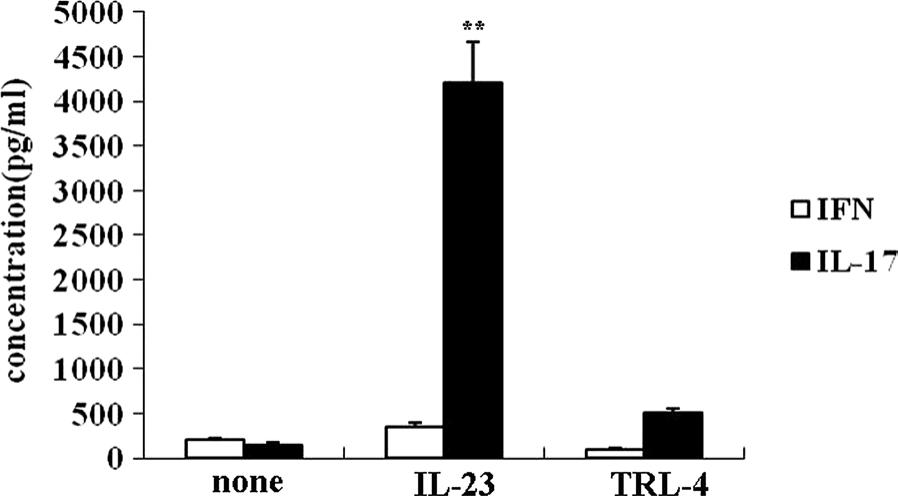
The capability of producing inflammatory cytokines by the Vδ1 γδ T cells was evaluated by detecting the concentration of IL-17 and IFN-γ in the supernatants of the cultured subset. The Vδ1 γδ T cells of PI-IBS patients significantly produced more IL-17 than control groups after stimulated by IL-23. All experiments were repeated three times. *p < 0.05, **p < 0.01

The function of the enriched Vδ1 γδ T cells was evaluated by proliferation assay, activation assay and pro-inflammatory cytokines production assay. Firstly, CCK-8 assay show that Vδ1 γδ T cells from PI-IBS patients significantly proliferated, with the absorbance OD value almost two times more than that from the control group (Fig. 4). Secondly, compared with the healthy human, the expression of CD62L molecule remarkably decreased while that of CD69 molecule increased (Fig. 5). The changes of the expression of these two molecules indicated that the cells’ activation occurred. Furthermore, stimulated with IL-23, the enriched Vδ1 γδ T cells from PI-IBS patients produced much more IL-17 but not IFN-γ, suggesting that the capability of producing pro-inflammatory cytokines, especially IL-17 of this subset boosted (Fig. 6). These results revealed the functional profile of Vδ1 γδ T cells in line with their quantitative changes in PI-IBS.

### Pro-inflammatory cytokines level in PI-IBS patients

**Fig. 7:**
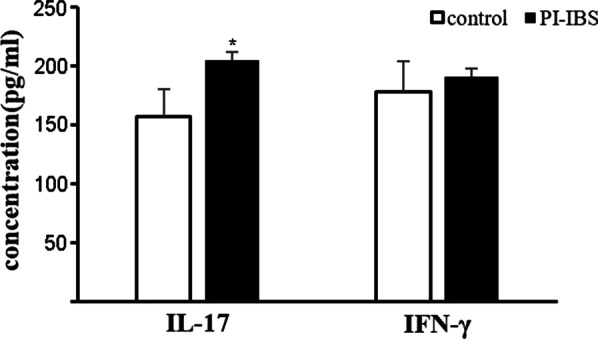
The concentration of IL-17 and IFN-γ in the peripheral blood was measured by ELISA. Compared with the control group, the peripheral IL-17 level significantly increased, suggesting the Vδ1 γδ T cells’ potential role. All experiments were repeated three times. *p < 0.05, **p < 0.01

**Fig. 8:**
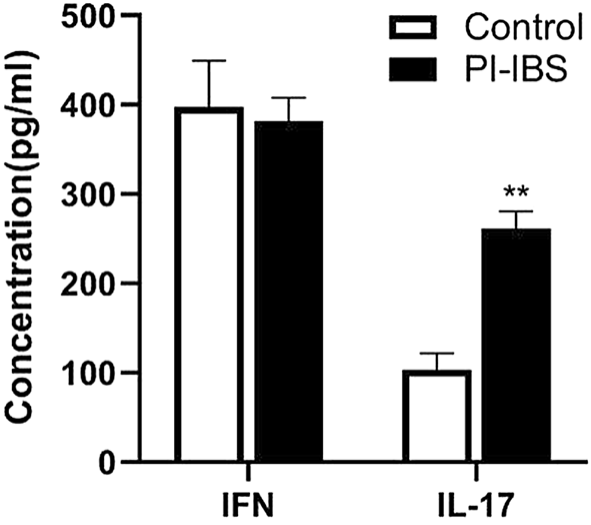
The concentration of IL-17 and IFN-γ in the colon tissue was measured by ELISA. Compared with the control group, the colonic IL-17 but not IFN-γ level significantly increased in PI-IBS. The change of pro-inflammatory cytokines in the patients accompanied with the increased and activated Vδ1 γδ T cells suggested that this kind of subset could participated in PI-IBS through producing IL-17. All experiments were repeated three times. *p < 0.05, **p < 0.01

The peripheral blood serum was collected and the colon tissue from PI-IBS was smashed by ultrosonic disintegrator. The IL-17 and IFN-γ concentration in the serum and tissue supernatants was measured by ELISA. As shown in Fig. 7, compared with the control group, the peripheral IL-17 level in PI-IBS patients significantly increased (*p* < 0.05) while IFN-γ remain unchanged (*p* > 0.05), suggesting that peripheral IL-17 could be involved in PI-IBS. As shown in Fig. 8, compared with the control group, the intestinal IL-17 level in PI-IBS patients significantly increased (*p* < 0.05), while the IFN-γ level remained unchanged (*p* > 0.05), suggesting that the local IL-17 could participate in the intestinal pathological disorder during PI-IBS. Furthermore, it was intriguing that the increase amplitude of IL-17 in colon tissue was far more than that in peripheral blood, implicating that the triggering and activating event did occur in the local intestine. All these results proved that during PI-IBS, IL-17 but not IFN-γ is the major proinflammatory cytokine.

## Discussion

Infection and inflammation are considered involved in the pathogenesis of IBS, whereas antibiotic therapy failed to induce expected improvement in all IBS patients, especially in those patients with refractory and prolonged symptoms [29]. This condition suggests that the immunity disturbance after infection could exert more crucial role in this disorder. γδ T cells participate in keeping the immune balance in the intestinal mucosa and associated with some digestive diseases [30–32]. Recently it is proved that γδ T cells present different function due to their various subsets’ distribution and function. For example, Costa et al. reported that Murine IL-17+ Vγ4 T lymphocytes accumulate in the lungs and play a protective role during servere sepsis. It is reported that murine IL-17+ V γ4 T lymphocytes accumulate in the lungs and play a protective role during servere sepsis., and that a novel proinflammatory human skin-homing Vγ9 Vδ2 T cell subset was identified with a potential role in psoriasis. The microbiota were associated with the development of γδ T cells subset. [33–36]. Thus it is important to investigate the precise role of the subset of γδ T cells in PI-IBS.

We found that the Vδ1 γδT cells was the predominant subset both in the peripheral blood and colon tissue. Usually in peripheral blood, Vδ2 γδT cells dominated but in PI-IBS condition, the major subset changed from Vδ2 to Vδ1. So where did the Vδ1 γδT cells come from? Firstly, the possible origin was intestine. In PI-IBS, the intestinal activated and proliferated Vδ1 γδ T cells traveled to the peripheral blood. Secondly, was other peripheral organ like lympho-node, spleen. Thirdly, they came directly from the the peripheral Vδ2 γδ T cells.

Did the subset polarity drifting have some functional meaning? We isolated and cultured Vδ1 γδ T cells subset, investigated their immune function in vitro. It is surprising that these Vδ1 γδ T cells subset from PI-IBS patients remarkably proliferated, activated and produced abundant IL-17. As for the pro-inflammatory cytokines, we found that the IL-17 level increased in the peripheral blood but IFN-γ remained unchanged, we also observed the similar phenomena as in the local intestine. These results suggested that the IL-17 both in peripheral and local intestine could come from Vδ1 γδ T cells. Interestingly, the increasing degree of IL-17 in peripheral blood was less than that in the local intestine. Because we cannot enrich Vδ1 γδ T cells from the patients’ intestine, we speculate that the local tissue microenvironment could promote the resident Vδ1 γδ T cells and contribute to the difference.

Sometimes the cells’ behavior in vivo could be different from that in vitro. Thus we explored the morphological alteration of the γδ T cells subset in the local colon from the PI-IBS patients. We found that Vδ1 γδ T cells significantly increased and Vδ2 γδ T cells decreased relatively. Simultaneously the IL-17 level in the colon tissue expanded. We previously reported that γδ T cells’Th17 response participated in the development of PI-IBS [28]. Thus our results suggested that Vδ1 γδ T cells could functionally is involved in Th17 response during PI-IBS.

An interesting problem is that whether the γδ T cells subset in peripheral blood share the same biological behavior with their counterpart in local colon tissue. Perhaps T lymphocyte homing assay could help to solve this puzzle problem. On the other hand, the γδ T cells subset could regulate each other via some unknown pathway. Probably Vδ2 γδ T cells participate in the pathological event with their own manner, not just as a bystander. Thus the precise role of the γδ T cell subset in the pathogenesis of PI-IBS needs an in-depth study.

There are also some shortcomings and limitations in our study. First of all, the number of samples used in this study is relative small and needs to be increased in further study. Although the low number of functional experiments is one limitation of our study, the results were very consistent between peripheral blood and local environment. Moreover, the deep molecular mechanisms of the regulation of γδ T cells on IL-17 need to be well investigated in future.

## Conclusions

In conclusion, the current study results show the pivotal role of Vδ1 γδ T cells and its stimulation product IL-17 in the development of PI-IBS. Various variable regions determine different immune response by γδ T cells subset in diverse pathological conditions. The Vδ1 γδ T cells are mainly presented in local mucosa tissue and Vδ2 γδ T cells were the majority in peripheral γδ T cells. In patients with post-infectious irritable bowel Syndrome (PI-IBS), we found that Vδ1 γδ T cells, instead of Vδ2 γδ T, dominated in both the peripheral blood and colonic tissue. Moreover, we also observed the increased ability of proliferation, activation and IL-17production of Vδ1 γδ T cells in PI-IBS patients after IL-23 stimulation. Taken together, these results suggest that Vδ1 γδ T cells was the predominant γδ T cells in both peripheral and intestinal tissue and was the major IL-17 producing γδ T cells in PI-IBS, which would be a novel therapeutic target for PI-IBS.

## Data Availability

The regarding supporting data and materials in our manuscript is available from the corresponding author.

## Abbreviations

PI-IBS: Post-infectious irritable bowel syndrome
IL-17: Interleukin 17
IFN-γ: Interferon gamma
mAb: Monoclonal antibody
FACS: Fluorescence activating cell sorter
TCR: T cell receptor
ELISA: Enzyme linked immunosorbent assay
